# Genome-scale characterization of the vacuole nitrate transporter *Chloride Channel* (*CLC*) genes and their transcriptional responses to diverse nutrient stresses in allotetraploid rapeseed

**DOI:** 10.1371/journal.pone.0208648

**Published:** 2018-12-20

**Authors:** Qiong Liao, Ting Zhou, Jun-yue Yao, Qing-fen Han, Hai-xing Song, Chun-yun Guan, Ying-peng Hua, Zhen-hua Zhang

**Affiliations:** 1 Southern Regional Collaborative Innovation Center for Grain and Oil Crops in China, College of Resources and Environmental Sciences, Hunan Agricultural University, Changsha, China; 2 National Center of Oilseed Crops Improvement, Hunan Branch, Changsha, China; ICAR-Indian Institute of Agricultural Biotechnology, INDIA

## Abstract

The *Chloride Channel* (*CLC*) gene family is reported to be involved in vacuolar nitrate (NO_3_^-^) transport. Nitrate distribution to the cytoplasm is beneficial for enhancing NO_3_^-^ assimilation and plays an important role in the regulation of nitrogen (N) use efficiency (NUE). In this study, genomic information, high-throughput transcriptional profiles, and gene co-expression analysis were integrated to identify the *CLCs* (*BnaCLCs*) in *Brassica napus*. The decreased NO_3_^-^ concentration in the *clca-2* mutant up-regulated the activities of nitrate reductase and glutamine synthetase, contributing to increase N assimilation and higher NUE in *Arabidopsis thaliana*. The genome-wide identification of 22*BnaCLC* genes experienced strong purifying selection. Segmental duplication was the major driving force in the expansion of the *BnaCLC* gene family. The most abundant cis-acting regulatory elements in the gene promoters, including DNA-binding One Zinc Finger, W-box, MYB, and GATA-box, might be involved in the transcriptional regulation of *BnaCLCs* expression. High-throughput transcriptional profiles and quantitative real-time PCR results showed that *BnaCLCs* responded differentially to distinct NO_3_^-^ regimes. Transcriptomics-assisted gene co-expression network analysis identified *BnaA7*.*CLCa-3* as the core member of the *BnaCLC* family, and this gene might play a central role in vacuolar NO_3_^-^ transport in crops. The *BnaCLC* members also showed distinct expression patterns under phosphate depletion and cadmium toxicity. Taken together, our results provide comprehensive insights into the vacuolar *BnaCLCs* and establish baseline information for future studies on *BnaCLCs*-mediated vacuolar NO_3_^-^ storage and its effect on NUE.

## Introduction

Nitrogen (N) is a fundamental non-mineral macronutrient, which is essential for the growth and development of higher plants [1]. China is the largest N-consuming country worldwide; although massive amounts of N are applied annually as fertilizer, crop yields are declining in some areas [2–3]. Hence, enhancing plant N use efficiency (NUE) is critical for developing sustainable agriculture [4]. Oilseed rape (*Brassica napus* L.) is a staple oil crop and has a high N requirement. To maintain its optimum yield, relatively high amounts of N fertilizer (from 150 to 300 kg N hm^-2^) are applied to soils [5–6], but only 30–50% of the applied N fertilizer is taken up from soil by crops [7]. The average NUE is approximately 35% in China, which results in N surpluses that are detrimental to the environment [4–5]. Therefore, the development of N-efficient cultivars through genetic improvement of crop NUE is a cost-effective and environmental friendly way to reduce excessive N in soils [8].

Inorganic nitrate (NO_3_^-^) is a predominant N-containing anion absorbed by upland crops, such as *B*. *napus*, under aerobic conditions [1]. NO_3_^-^ uptake, accumulation, and utilization have been reported to have close relationships with NUE [2, 9, 10, 11]. Four NO_3_^-^ transporters, namely nitrate transporter 1 (NRT1), nitrate transporter 2 (NRT2), chloride channel (CLC) family proteins, and slow-type anion channel associated homologs (SLAC1/SLAH1-4), have been implicated in efficient N uptake and transport [12]. To cope with fluctuating NO_3_^-^ concentrations in soils, NRT1 and NRT2, which are low-affinity (Km = 0.5 mM) and high-affinity (Km = 10–100 mM) transport systems, respectively, work together to ensure efficient NO_3_^-^ uptake [13–14]. Once it has entered the roots, NO_3_^-^ can be stored *in situ* or undergo long-distance transport to shoots where it can be assimilated or stored in vacuoles.

The 2NO_3_^-^/H^+^ antiporter *AtCLCa* responsible for NO_3_^-^ storage in vacuoles first identified from *Arabidopsis thaliana* (*A*. *thaliana*) cells belongs to the *CLC* gene family [15]. Members of this family specifically transport chloride as Cl^-^/H^+^ antiporters or channels [16–17]. Geelen et al. (2000) found that the *clca-1* mutant presented reduced NO_3_^-^ content, and then De Angeli et al. (2006) first demonstrated that *AtCLCa* is a tonoplast-localized NO_3_^-^ antiporter that is involved in the regulation of NO_3_^-^ sequestration into vacuoles [15, 18]. Three of the six other members in the *CLC* gene family, namely *AtCLCb*, *AtCLCc*, and *AtCLCg* [19], are also localized to tonoplasts [20]. Although *AtCLCb* functions as a 2NO_3_^-^/H^+^ antiporter in *Xenopus oocytes*, it is still unknown if it is involved in vacuolar NO_3_^-^ storage in plants [20]. While *AtCLCc* seems to be responsible for NO_3_^-^ and chloride homeostasis, and essential for stomatal movement and salt tolerance by regulating chloride transport [21–22], *AtCLCg* participates in salt tolerance by altering chloride homeostasis in mesophyll cells [23]. Both the AtCLCd and AtCLCf proteins are localized to Golgi membranes. However, *AtCLCd* plays a crucial role in the regulation of luminal pH in the trans-Golgi network by transporting chloride or NO_3_^-^ [24], whereas the function of *AtCLCf* remains elusive [25]. *AtCLCe* is situated in the thylakoid membrane of chloroplasts and participates in the regulation of photosynthetic electron transport [26].

*B*. *napus* is a staple oil crop worldwide. The allotetraploid *B*. *napus* (A_n_A_n_C_n_C_n_, ~1345 Mb, 2n = 4x = 38) was derived from the natural hybridization between *Brassica rapa* (A_r_A_r_, ~485 Mb, 2n = 2x = 20) [27] and *Brassica oleracea* (C_o_C_o_, ~630 Mb, 2n = 2x = 18) [28] ~7,500 years ago [29–31]. The allopolyploidy events occurring in *B*. *napu*s resulted in more duplicated segments and homologous regions within the rapeseed genome [30] than those found in *A*. *thaliana* (~125 Mb, 2n = 2x = 10) (Arabidopsis Genome Initiative 2000), which also belongs to the Brassicaceae family.

*B*. *napus* has a higher requirement than other cereals for N-containing nutrients to maintain its optimal growth [8]. In rice, overexpression of *OsNRT2*.*3b* contributes to enhance NO_3_^-^ uptake and grain yield in the field [32]. In *A*. *thaliana*, mutation of *AtCLCa* was shown to decrease NO_3_^-^ concentrations in tissues and improve NUE [4]. However, the genome-wide organization of vacuolar NO_3_^-^ transporter genes in *B*. *napus*, especially the *CLC* gene family, is poorly understood. Thus, in the present study, we aimed to provide: (i) a genome-wide identification of the *CLC* family members in *Brassica* sp. crops; (ii) a comprehensive analysis of the molecular characteristics of the tonoplast-localized NO_3_^-^ transporter genes in *B*. *napus*; and (iii) the identification of the core members of the *BnaCLC* gene family and their transcriptional responses to different N regimes. The genome-wide identification and molecular characterization of the *BnaCLC* family members presented here will provide fundamental information for NUE improvement and for further research on the biological functions and evolutionary relationships within this gene family in *B*. *napus* and other crop species.

## Materials and methods

### Plant materials and growth conditions

The *B*. *napus* cultivar ‘Xiang-you 15’ (XY15) was provided by the Improvement Center of National Oil Crops (Hunan Branch, Changsha, Hunan Province, China).*A*. *thaliana* wild-type Wassilewskija (Ws) was used as control for the chloride channel accessory 2 (*clca-2*) mutant (FST 171A06), as described in De Angeli et al. (2006) [15]. Both plant lines were obtained from the Institute National de la Recherche Agronomique collection in France.

All plants were grown in an incubator set at 70% relative humidity, 16-h-light/8-h-dark cycle, and a constant temperature of 22°C. Plant lines were sowed in a matrix consisting of vermiculite and perlite at a ratio of 3:2. After germination, *B*. *napus* were transplanted and hydroponically grown in plastic boxes (2 L), as described by Han et al. (2016) [4]. These boxes were arranged in a completely randomized design with three biological replicates. The Hoagland nutrient solution (pH 5.8) provided to plants was replaced every 3 days. As described in Han et al. (2016) [4], Ws and *clca-2* were transplanted and hydroponically grown in plastic boxes (550 mL), and the nutrient solution was replaced every 5 days. These boxes were arranged in a completely randomized design with four biological replicates.

### Determination of NO_3_^-^ and N concentrations

As described by Han et al. (2016) [4], plant tissues were sampled, bathed in boiling water for 30 min, and then assayed forNO_3_^-^ content using a continuous-flow auto-analyzer (AA3, Seal Analytical Inc., Southampton, UK). The whole seedlings of hydroponically grown *A*. *thaliana* were sampled and oven dried at 105°C for 30 min, and then at 65°C until they reached a constant weight. N concentrations were determined using the Kjeldahl method [33], Total N = N concentration × biomass. The NUE value based on biomass was calculated based on the following formula: NUE = total biomass/ total N.

### Determination of the activities of nitrate reductase (NR) and glutamine synthetase (GS)

The method for determining NR activity was slightly modified from that presented in previous studies [34–35]. Five milliliters of phosphate buffer (0.1M, pH7.5) was added to frozen root tissue samples (~0.3 g) and then ground to homogeneity using a chilled mortar and pestle in the presence of acid-washed sand. The homogenates were centrifuged at 2,000 ×g for 15 min at 4°C, and the resulting supernatant was assayed for NADH-dependent NR activity. The reaction mixture consisted of 0.4 mL supernatant, 0.1 M KNO_3_, and 3 mM NADH. The reaction was terminated after 30 min at 25°C by the addition of 1% sulfanilamide and α-naphthylamine. The amount of reaction product was measured at 540 nm using the UV-2600 spectrophotometer (Shimadzu Corp., Kyoto, Japan).

The activity of GS was determined by quantification of γ-glutamyl hydroxamate as described by Culimore and Sims. (1980) [36]. Five milliliters of Tris-HCl buffer (0.05M Tris-HCl, 0.5 mM MgCl_2_, 1.0mM EDTA, pH7.8) was added to frozen root tissue samples (~0.3 g) and then ground to homogeneity using a chilled mortar and pestle. The homogenates were centrifuged at 8,000 ×g for 15 min at 4 °C, and the resulting supernatant was used to analyze GS activity. The reaction mixture contained 1.2 mL supernatant, 0.25M imidazole buffer (pH 7.0), 0.3 M sodium glutamate, 0.3 mM ATP, 0.5M MgSO_4_, and 2 M hydroxylamine, and it was incubated for 15 min at 25°C. An acidic FeCl_3_ solution (0.088 M FeCl_3_, 0.67 M HCl, and 0.20 M trichloroacetic acid) was added to terminate the reaction, and the amount of Fe (III)-complex in the reaction product (γ-glutamyl hydroxamate) was measured at 540 nm using the UV-2600 spectrophotometer (Shimadzu Corp.).

### Retrieval of the *CLC* family gene sequences

The *CLC* genes of *Brassica* species were identified based on their similarities to *A*. *thaliana* homologs. The genomic sequences, coding sequences (CDS), protein sequences, and gene IDs of the seven *CLC* genes (*CLCa–g*) were obtained from the Arabidopsis Information Resource (TAIR, https://www.arabidopsis.org/) database. We used the *CLC* gene sequences of *A*. *thaliana* as the seed sequences, and performed a basic local alignment search tool analysis using protein sequences as queries (BLASTp) to retrieve homologous gene sequences with an E value < 1e^-10^ in *Brassica* crops and other species. The databases used included The *Brassica* Database (BRAD) Version 1.1 (http://brassicadb.org/brad/) [37] for *B*. *rapa*, *Bol* Base Version 1.0 (http://119.97.203.210/bolbase/index.html) for *B*. *oleracea* [38], Genoscope (http://www.genoscope.cns.fr/brassicanapus/) for *B*. *napus* [30], and the National Center for Biotechnology Information (NCBI, www.ncbi.nlm.nih.gov) for other species [39]. In the present study, genes from *Brassica* species were named as follows: (abbreviation of the species name) + (chromosome number) + (period) + (name of gene homolog in *A*. *thaliana*). For example, *BnaC6*.*CLCa-1* represents the *CLCa* gene on chromosome C6 of *B*. *napus*. All the data were retrieved on March 10, 2018.

### Chromosomal localization of the CLC family genes in *B*. *napus*

The starting positions of all the *CLC* family genes in *B*. *napus* were obtained from BRAD using the complete nucleotide sequences of *B*. *napus*. The MapInspect software (http://www.softsea.com/review/MapInspect.html) was then used to draw chromosomal location diagrams for these genes.

### Multiple alignment and phylogenetic analysis of the BnaCLC family proteins

Multiple sequence alignments of the CLC protein sequences were performed using the ClustalW tool of MEGA 6.06 [40]. Based on these alignments, an unrooted phylogenetic tree, comprising 128 full-length CLC protein sequences, was constructed in MEGA 6.06 using the neighbor joining method, and Poisson correction, pairwise deletion, and bootstrapping (1000 replicates; random seeds) as the required parameters.

### Characterization of conserved motifs and physiochemical characteristics of the BnaCLC family proteins

The protein sequences of *A*. *thaliana* and *Brassica* species were submitted to the Multiple Expectation maximization for Motif Elicitation (MEME) online software (http://meme-suite.org/tools/meme, Version 4.11.2) for characterizing the conserved motifs/domains [41]. Default parameters were generally used, except for the optimum motif width, which was set to 6–50 bp, and the maximum number of motifs, which was set to 10. The ExPASy ProtoParam (http://www.expasy.org/tools/protparam.html) [42] tool was used to obtain the number of amino acids, molecular weights (MWs, kDa), theoretical isoelectric points (pIs), grand average of hydropathy (GRAVY) values, and instability indexes (a protein with instability index > 40 was considered unstable) [43].

### Evolution selection pressure analysis and functional divergence of the *BnaCLC* family genes

Pairwise alignments of gene CDS without stop codons were performed in ClustalW (http://www.clustal.org/clustal2/) [44], and then subject to the KaKs_Calculator toolkit (https://sourceforge.net/projects/kakscalculator2/) [27] to obtain the non-synonymous substitution rate (Ka), synonymous substitution rate (Ks), and Ka/Ks values by implementing the yn00 method [45]. The formula T = Ks/2λ (where λ = 1.5 × 10^−8^ for Brassicaceae) [46] was used to calculate the probable age of segmental duplication. The Detecting Variability in Evolutionary Rates among Genes software (DIVERGE) 3.0 (http://xungulab.com/software/diverge3/diverge3.html) [47] was used to detect gene functional divergence through multiple alignments of CDS and protein sequences.

### Exon-intron structure of the *BnaCLC* family genes

The full-length CDS and genomic sequences of the *CLC* family genes were submitted to the online Gene Structure Display Server (GSDS) 2.0 (http://gsds.cbi.pku.edu.cn/) [48] to analyze their exon-intron structures.

### Identification of putative cis-acting regulatory elements (CREs) in the *BnaCLC* gene promoters

A 2.0-kb fragment of each genomic sequence upstream of the start codon (ATG) was downloaded from the *Brassica napus* Genome Browser (http://www.genoscope.cns.fr/brassicanapus/) [30]. These sequences were submitted to PLACE 30.0 (http://www.dna.affrc.go.jp/PLACE/) [49] to examine putative CREs. The distributions of the over-presented CREs along the promoters were displayed in PROSITE (https://prosite.expasy.org/mydomains/). The enriched CREs were displayed using the word cloud generator WordArt (https://wordart.com/).

### Transcriptional profiling and identification of core gene members within the *BnaCLC* family

The *B*. *napus* cultivar XY15 was used to identify the genome-wide mRNA transcriptome responses of this species to NO_3_^-^ depletion and replenishment conditions. The hydroponic grown *B*. *napus* seedlings were cultivated and processed according to the method described by Han et al. (2016) [4]. Briefly, *B*. *napus* seedlings were cultivated under high NO_3_^-^ solutions (9.0 mM) for 9 days and then transferred to NO_3_^-^-free solutions for 3 days. For NO_3_^-^-depletion treatments, the seedlings were cultivated in NO_3_^-^-free solutions (control). For NO_3_^-^-replenishment treatments, the seedlings were transferred to high NO_3_^-^ (9.0 mM) for 6 h. The shoots and roots of the rapeseed seedlings subject to each treatment were then sampled. Each sample, including three independent biological replicates, was then used for high-throughput transcriptome profiling.

Gene co-expression analysis was used to identify gene interactions and the core genes within the *BnaCLC* family members. Default thresholds of *Pearson* correlation values were set (http://plantgrn.noble.org/DeGNServer/Analysis.jsp), and CYTOSCAPE 3.2.1 was used to construct the gene co-expression networks [50].

### Transcriptional responses of the *BnaCLC* members under cadmium (Cd) toxicity and inorganic phosphate (Pi) depletion

Quantitative reverse-transcription PCR (qRT-PCR) was used to identify the relative gene expression of the *BnaCLCa* members. For the Cd toxicity treatment, the rapeseed plants were hydroponically cultivated in a Cd-free solution for 10 d, and then transferred to the 10 μM CdCl_2_ treatment for 6 h. For the inorganic phosphate (Pi) starvation treatment, the rapeseed seedlings were first grown under 250 μM Pi (KH_2_PO_4_) for10 d, and then transferred to the a Pi-free solution for 5 d. The shoots and roots were individually harvested and stored at -80 °C until RNA isolation.

After treatment with RNase-free DNase I, the total RNAs isolated from samples were used as templates for cDNA synthesis with the PrimeScript RT reagent Kit with gDNA Eraser (Perfect Real Time) (TaKaRa, Shiga, Japan). The qRT-PCR assays for the detection of relative gene expression were performed using SYBR Premix Ex Taq II (TliRNaseH Plus) (TaKaRa) under an Applied Biosystems StepOne Plus Real-time PCR System (Thermo Fisher Scientific, Waltham, MA, USA). The thermal cycles were as follows: 95°C for 3 min, followed by 40 cycles at 95°C for 10 s and 60°C for 30 s. Melting curve analysis to ensure primer specificity was conducted as follows: 95°C for 15 s, 60°C for 1 min, 60–95°C for 15 s (+0.3°C per cycle). Expression data were normalized using the public reference genes *BnaEF1-α* [51] and *BnaGDI1* [52], and relative gene expression was calculated with the 2^-ΔΔC*T*^ method [53].

### Statistical analysis

Significant differences (*P*-value < 0.05) were determined by one-way analysis of variance(ANOVA), followed by Tukey’s honestly significant difference (HSD) multiple comparison tests, using the Statistical Package for the Social Sciences17.0 (SPSS, Chicago, IL, USA).

### Availability of data and materials

We have upload the raw high-throughput sequencing data as supporting information files (S3 Table), in addition, rapeseed seeds that are required to reproduce these findings can be shared by contacting the corresponding author, Zhen-hua Zhang (zhzh1468@163.com) or Ying-peng Hua (yingpenghua89@126.com)

## Results

### Decreased vacuolar sequestration capacity (VSC) of NO_3_^-^ results in enhanced NUE in the *clca-2* mutant

**Fig 1:**
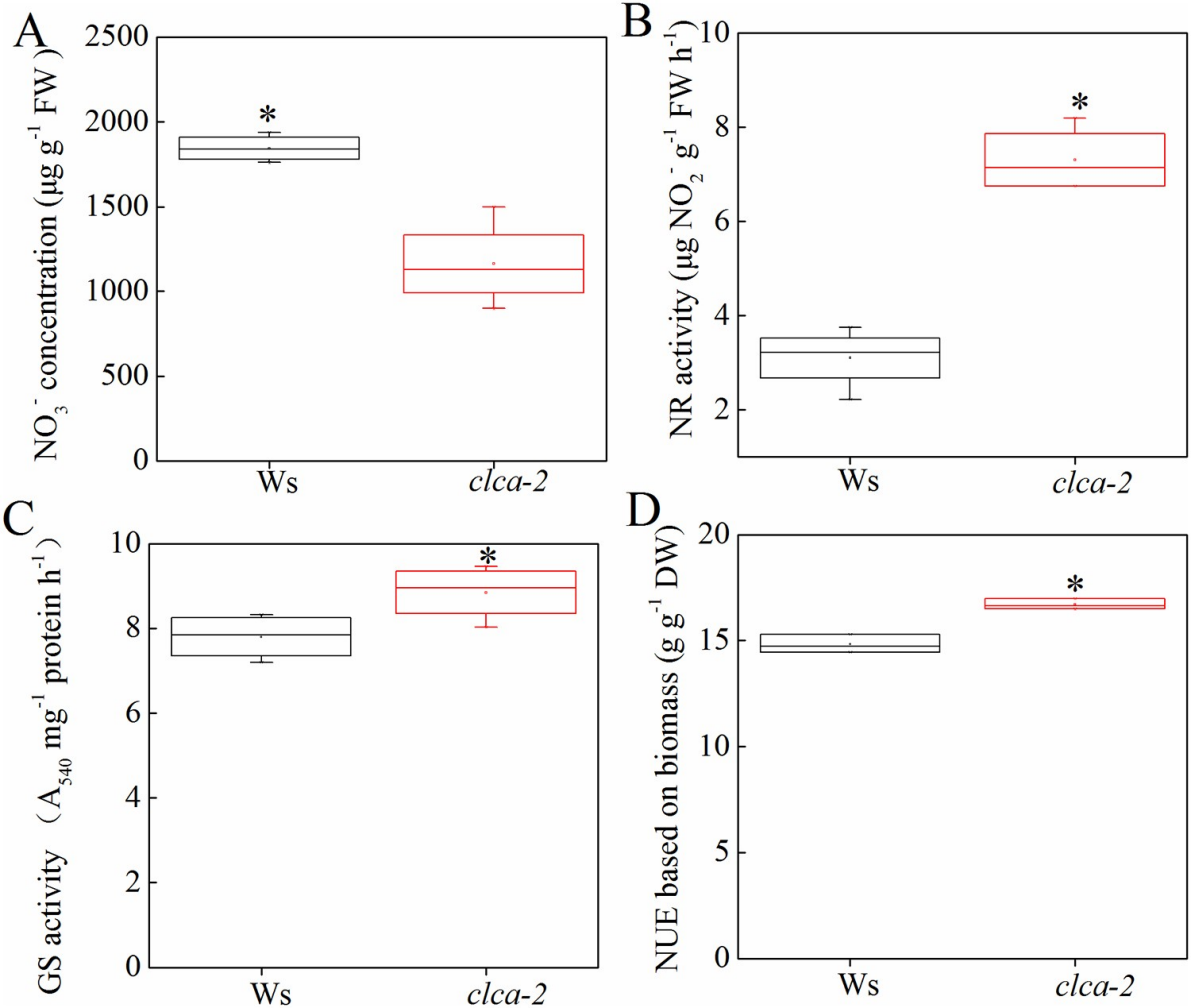
Decreased NO_3_^-^ concentration in the *clca-2* mutant accelerated nitrogen (N) assimilation, resulting in higher N use efficiency (NUE). Plants grown hydroponically were sampled for further analysis at the seedling stage. Conditions for hydroponics culture and characteristics of *Arabidopsis thalina (A*. *thaliana)* are defined in the “Methods”. The wild-type Wassilewskija (Ws) plants were used as the control for the *CLC* null mutant (*clca-2*). At the seedling stage, plants were sampled for NO_3_^-^ assays (A), and the activities of nitrate reductase (NR) (B) and glutamine synthetase (GS) (C) were shown in Ws and *clca-2*. NUE values were calculated based on total biomass (shoot and root) per total N uptake, and the differences in NUE of Ws and *clca-2* were shown in D. Bars indicate standard deviations (SD) of four biological replicates. The asterisks denote significant differences at *P* < 0.05.

*AtCLCa* has been defined as a 2NO_3_^-^/H^+^ antiporter and participates in NO_3_^-^ storage in vacuoles [15]. To investigate the effect of impaired NO_3_^-^ VSC on NUE in the *clca-2* mutant, we first determined the NO_3_^-^ concentrations in both Ws and *clca-2* plants. The results showed that NO_3_^-^ concentration was significantly lower in the *clca-2* mutant than in Ws (Fig 1A). Further, the activities of some key enzymes involved in N assimilation, namely NR and GS, were determined. They were significantly higher in the *clca-2* mutant than in Ws (Fig 1B and 1C). These results suggested that there was more NO_3_^-^ in the cytoplasm of the *clca-2* mutant than in the cytoplasm of Ws, which induced the activities of NR and GS thereby contributing to enhance N assimilation and NUE (Fig 1D). Thus, the decreased NO_3_^-^ VSC regulated by *AtCLCa* improved NUE in *A*. *thaliana*. However, a genome-wide analysis of the *CLC* gene family in *B*. *napus* was necessary to further explore the functions of these genes and to provide a theoretical basis for studying their effects on the NUE of this species.

### Comparative analysis and phylogenetic relationships among the *BnaCLC* family genes

The sequences and gene IDs of the seven *AtCLCs* genes were used to perform BLAST searches against the genomes in the *Brassica* database. We found that *B*. *rapa*, *B*. *oleracea*, and *B*. *napus* had 11, 10, and 22 *CLC* homologs, respectively (S1 Table). This indicated that the *BnaCLCs* did not suffer gene loss or addition during the allopolyploidy process of the rapeseed genome (A_n_A_n_C_n_C_n_, 2n = 4x = 38). Similar to *A*. *thaliana*, the *CLC* genes identified in the three *Brassica* species can also be divided into seven subgroups (*CLCa–g*). Specifically, the 22 *BnaCLC* family genes comprised four *BnaCLCas*, four *BnaCLCbs*, four *BnaCLCcs*, two *BnaCLCds*, two *BnaCLCes*, four *BnaCLCfs*, and two *BnaCLCgs*.

**Fig 2:**
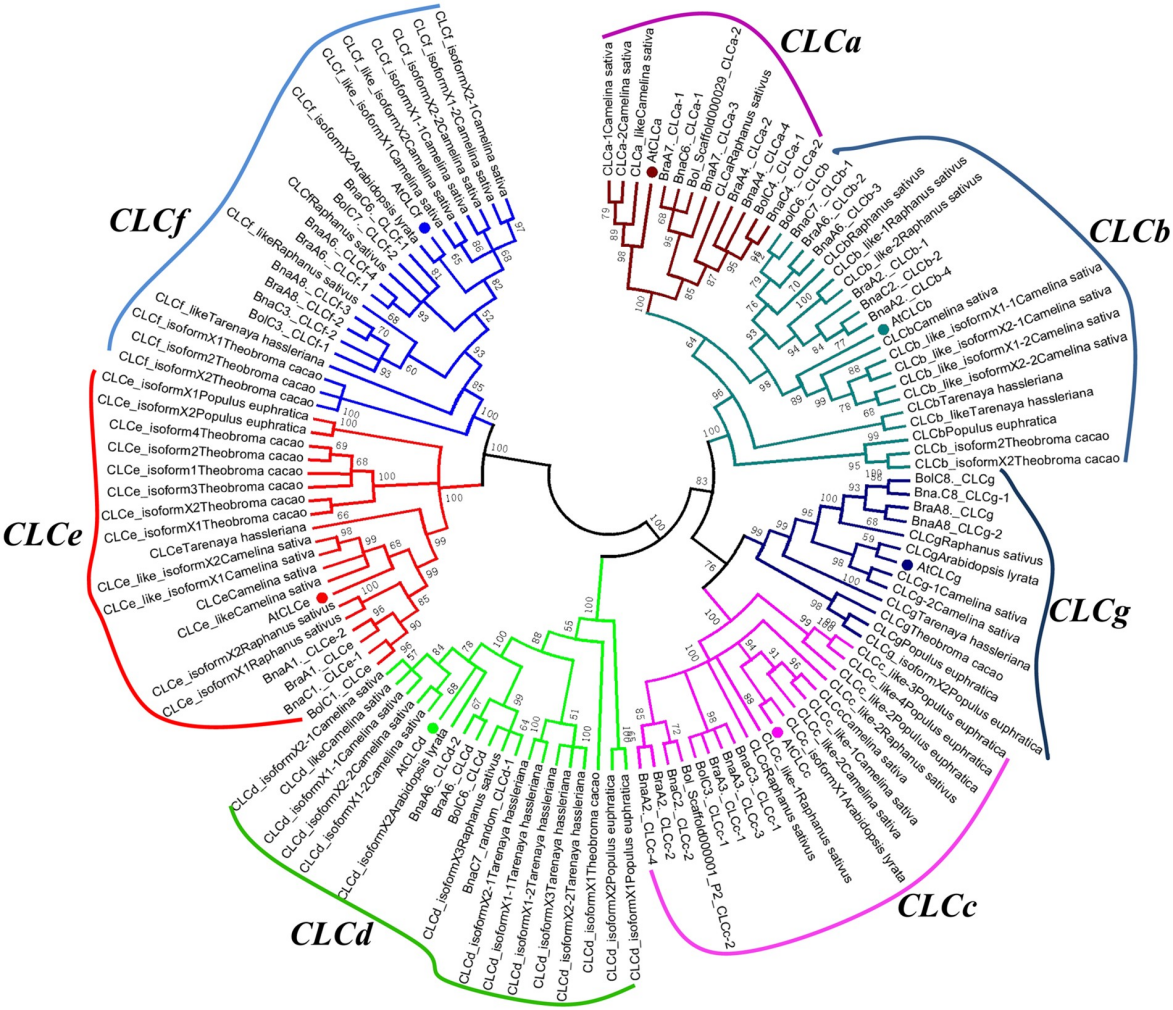
Phylogenetic tree of *CLCs* in diverse plant species. A total of 118 protein sequences from 10 species including *Brassica napus*, *Brassica rapa*, *Brassica oleracea*, *Arabidopsis thaliana*, *Arabidopsis lyrata*, *Camelina sativa*, *Populus euphratica*, *Raphanus sativus*, *Tarenaya hassleriana* and *Theobroma cacao* were multi-aligned using the ClustalW program, and then an unrooted phylogenetic tree was constructed using the software MEGA6.06 with the neighbor-joining method. The percentage of replicate trees, in which the associated taxa clustered together in the bootstrap test (1000 replicates), are shown next to the branches.

To determine the molecular evolution and phylogenetic relationships among the CLC proteins from several species, an unrooted phylogenetic tree was constructed based on the amino acid sequences of 128 *CLC* genes from 10 plant species (Fig 2): the four *Brassica* species plus *Arabidopsis lyrata*, *Camelina sativa*, *Populus euphratica*, *Raphanus sativus*, *Tarenayahassleriana* and *Theobroma cacao*. The *CLC* family genes were classified into seven clades regardless of plant species (Fig 2), which is consistent with the subfamily categorization. This indicated that *CLC* protein divergence occurred before organism speciation. Furthermore, in *Brassica* species, each subfamily member of the *CLC* family was closely clustered with the corresponding homologs in *A*. *thaliana* (Fig 2). Most of the *CLC* proteins within each subfamily had very short branch lengths (Fig 2), indicating a recent genetic divergence.

### Chromosomal localization and gene expansion patterns of the *BnaCLC* genes

**Fig 3:**
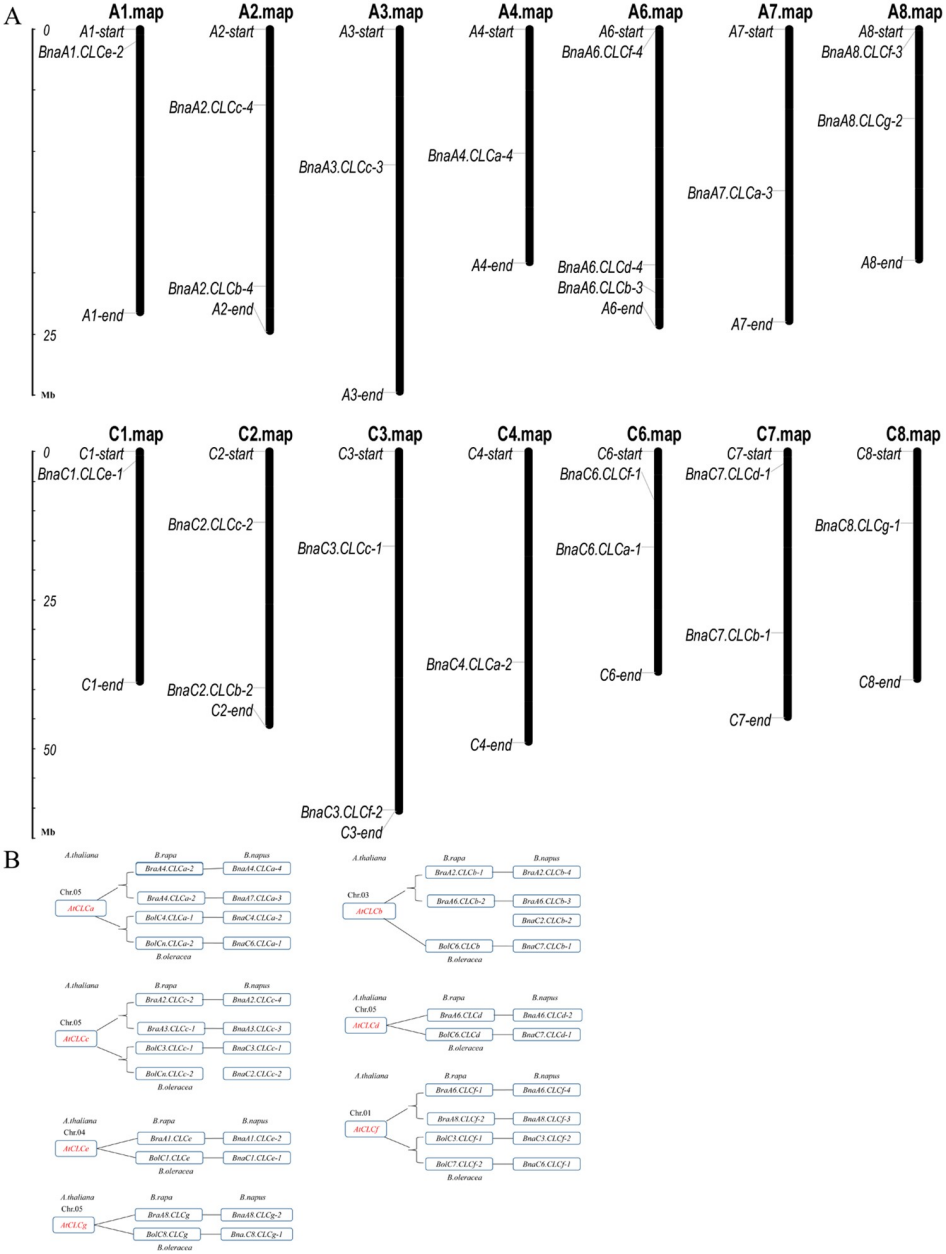
Chromosomal location and expansion events of the *CLC* gene family in *B*. *napus*. The starting positions of all *BnaCLCs* were collected from BRAD through BLASTn search, and the gene chromosomal location diagram was drawn using the MapInspect software (A). Evolutionary processes and expansion events of the *CLC* gene family in *B*. *napus* (B). The homologues between different Brassica species are connected by lines.

The *CLC* genes in *B*. *napus* were physically mapped onto 14 chromosomes (A_n_ subgenomes: A_n_1, A_n_2, A_n_3, A_n_4, A_n_6, A_n_7, and A_n_8; C_n_ subgenomes: C_n_1, C_n_2, C_n_3, C_n_4, C_n_6, C_n_7, and C_n_8). All *BnaCLCs* were evenly distributed on each of the A_n_ (11) and C_o_ (11) genomes (Fig 3A). Comparative genomics revealed that the *A*. *thaliana* genome is divided into 24 ancestral crucifer blocks (labeled A-X) [54]. Here, the members of the seven *CLC* subfamilies were mapped to six blocks (Table 1). All the *BnaCLCa* and *BnaCLCg* members were mapped to the S block, and the members belonging to the other five *CLC* subfamilies were located within the L, Wb, Q, U, and C blocks, respectively (Table 1). The *Brassica* genomes resulting from whole genome triplication are separated into least fractionated (LF), moderate fractionated (MF1), and most fractionated (MF2) subgenomes [55]. There were 43 *CLCs* in *B*. *rapa*, *B*. *oleracea*, and *B*. *napus*. Approximately 37.2% (16) of the *CLCs* belonged to the LF subgenome, and the MF1 and MF2 subgenomes comprised 17 and 10 *CLC* family genes, accounting for 39.5% and 23.3%, respectively (Table 1). To further understand the *CLC* gene expansion patterns, we investigated gene duplication events in *B*. *napus*, which revealed that segmental duplication played an important role in the expansion of the *BnaCLC* family genes (Fig 3B).

**Table 1:** Molecular characterization of the *BnaCLC* family proteins in *Brassica* crops (*B*. *rapa*, *B*. *oleracea* and *B*. *napus*).

Gene name	Gene ID	Block	Subgenome	CDS (bp)	Amino acids	MW (kD)	pI	Instablility index	GRAVY
*BnaC6*.*CLCa-1*	BnaC06g13300D	S	MF1	2313	770	84.74	8.14	36.55	0.250
*BnaC4*.*CLCa-2*	BnaC04g33870D	S	MF1	2331	776	85.36	8.11	36.40	0.264
*BnaA7*.*CLCa-3*	BnaA07g15180D	S	MF1	2313	770	84.67	7.91	36.76	0.257
*BnaA4*.*CLCa-4*	BnaA04g11860D	S	MF2	2331	776	85.43	7.89	36.60	0.256
*BraA7*.*CLCa-1*	Bra028478	S	MF1	2313	770	84.74	8.14	36.90	0.253
*BraA4*.*CLCa-2*	Bra030328	S	MF2	2331	776	85.44	8.11	36.60	0.256
*BolC4*.*CLCa-1*	Bol006752	S	MF1	2331	776	85.36	8.11	36.40	0.264
*BolCn*.*CLCa-2*	Bol036409	S	MF1	2313	770	84.74	8.14	36.55	0.250
*BnaC7*.*CLCb-1*	BnaC07g24030D	L	LF	2352	783	86.28	7.55	37.77	0.254
*BnaC2*.*CLCb-2*	BnaC02g36720D	L	MF1	2352	783	86.10	8.64	34.02	0.305
*BnaA6*.*CLCb-3*	BnaA06g32380D	L	LF	2352	783	86.47	7.27	38.22	0.240
*BnaA2*.*CLCb-4*	BnaA02g28670D	L	MF1	2343	780	85.86	8.72	33.57	0.284
*BraA2*.*CLCb-1*	Bra032985	L	MF1	2352	783	86.12	8.65	33.28	0.291
*BraA6*.*CLCb-2*	Bra025253	L	LF	2352	783	86.44	7.27	38.11	0.240
*BolC6*.*CLCb*	Bol042862	L	LF	2352	783	86.29	7.55	38.36	0.254
*BnaC3*.*CLCc-1*	BnaC03g27500D	Wb	MF1	2328	775	84.43	8.77	38.12	0.390
*BnaC2*.*CLCc-2*	BnaC02g16210D	Wb	MF2	2328	775	84.33	8.55	36.91	0.396
*BnaA3*.*CLCc-3*	BnaA03g23270D	Wb	MF1	2328	775	84.47	8.77	38.01	0.389
*BnaA2*.*CLCc-4*	BnaA02g11800D	Wb	MF2	2328	775	84.41	8.55	36.91	0.396
*BraA3*.*CLCc-1*	Bra000596	Wb	MF1	2328	775	84.44	8.77	38.12	0.386
*BraA2*.*CLCc-2*	Bra022507	Wb	MF2	2328	775	84.41	8.55	36.91	0.396
*BolC3*.*CLCc-1*	Bol015059	Wb	MF1	2328	775	84.43	8.77	38.12	0.390
*BolCn*.*CLCc-2*	Bol045308	Wb	MF2	2328	775	84.36	8.55	36.75	0.396
*BnaC7*.*CLCd-1*	BnaC07g49590D	Q	LF	2409	802	88.15	8.55	41.83	0.217
*BnaA6*.*CLCd-2*	BnaA06g28170D	Q	LF	2379	792	87.10	8.35	42.76	0.214
*BraA6*.*CLCd*	Bra009891	Q	LF	2379	792	87.10	8.35	42.76	0.214
*BolC6*.*CLCd*	Bol022298	Q	LF	2409	802	88.13	8.55	41.39	0.218
*BnaC1*.*CLCe-1*	BnaC01g03130D	U	LF	2142	713	75.30	5.18	50.75	0.293
*BnaA1*.*CLCe-2*	BnaA01g01990D	U	LF	2106	701	74.08	5.52	50.99	0.315
*BraA1*.*CLCe*	Bra011615	U	LF	2103	700	74.03	5.35	53.74	0.306
*BolC1*.*CLCe*	Bol029074	U	LF	3096	1031	110.71	5.56	46.06	0.164
*BnaC6*.*CLCf-1*	BnaC06g07440D	C	LF	2346	781	83.71	6.46	40.09	0.019
*BnaC3*.*CLCf-2*	BnaC03g70680D	C	MF1	2298	765	81.95	7.57	43.97	0.091
*BnaA8*.*CLCf-3*	BnaA08g00400D	C	MF1	2292	763	81.90	6.80	43.86	0.071
*BnaA6*.*CLCf-4*	BnaA06g00250D	C	LF	2358	785	83.99	6.28	40.90	0.021
*BraA6*.*CLCf-1*	Bra038007	C	LF	2358	785	83.98	6.28	41.14	0.023
*BraA8*.*CLCf-2*	Bra030845	C	MF1	2331	776	83.28	6.31	43.48	0.057
*BolC3*.*CLCf-1*	Bol035224	C	MF1	2355	784	83.94	6.22	41.72	0.059
*BolC7*.*CLCf-2*	Bol039017	C	LF	2346	781	83.69	6.46	40.09	0.022
*BnaC8*.*CLCg-1*	BnaC08g08120D	S	MF2	2295	764	83.90	8.82	41.49	0.461
*BnaA8*.*CLCg-2*	BnaA08g07280D	S	MF2	2295	764	83.92	8.86	41.75	0.468
*BraA8*.*CLCg*	Bra038948	S	MF2	2295	764	83.90	8.86	41.75	0.465
*BolC8*.*CLCg*	Bol027031	S	MF2	2295	764	83.84	8.75	40.96	0.463

Note: CDS, coding sequence; MW, molecular weight; pI, isoelectric point; GRAVY, grand average of hydropathy; LF, Least fractionated subgenome; MF1, medium fractionated subgenome; MF2, more fractionated genome.

### Conserved domain analysis of the *CLC* family genes in *Brassica* species

**Fig 4:**
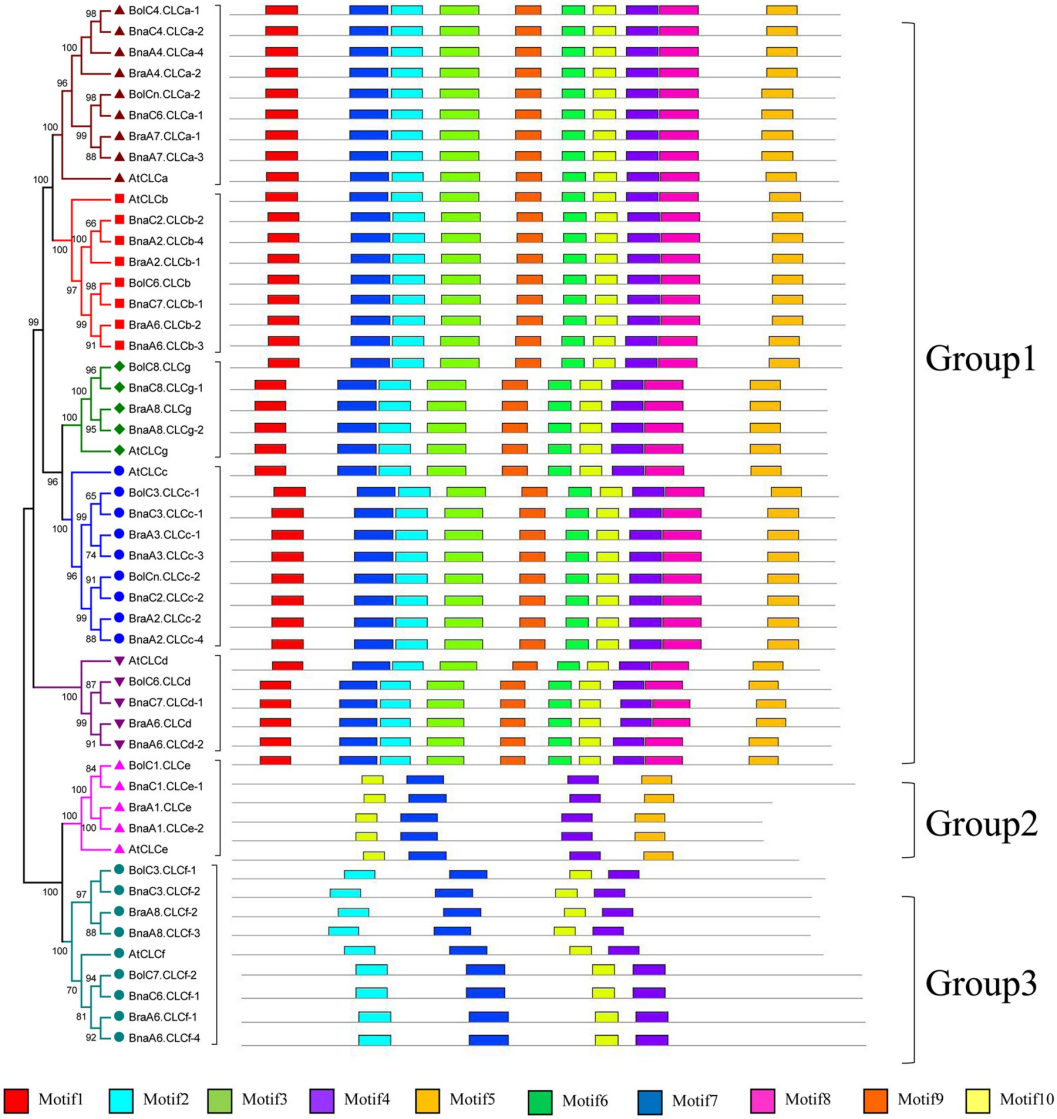
Characterization of the conserved motifs in the CLC proteins in *A*. *thaliana* and Brassica crops (*B*. *rapa*, *B*. *oleracea* and *B*. *napus*). Conserved motifs were predicted by the MEME program. The boxes with different color represent different conserved motifs, and the gray lines indicate non-detected motifs in the *CLC* family genes.

To characterize the conserved motifs in the *CLC* gene family, the MEME program was used to align the protein sequences of the *CLC* family genes. As shown in Fig 4, the *CLC* family genes were divided into three groups according to the classification of their conserved motifs. Group 1 comprised five subfamilies, namely *CLCa*, *CLCb*, *CLCc*, *CLCd*, and *CLCg*, which contained all of the 10 motifs predicted (S1A Fig). The *CLCf* and *CLCe* subfamilies were clustered in Group 2 and Group 3, respectively, and only four motifs were found in these two groups. All the *CLC* genes of *Brassica* species contained Motifs 4/7/10 (S1B Fig), demonstrating that these three motifs are highly conserved and might be used as markers for the identification and characterization of *CLC* genes in these crops. To further characterize these three highly conserved motifs, their short amino acid sequences were analyzed in WebLogo.

### Physicochemical characteristics of the *CLC* family genes and proteins in Brassica species

As shown in Table 1, the full-length CDS of the *CLC* genes in *Brassica* species range from 2,103 bp (*BraA1*.*CLCe*) to 3,096 bp (*BolC1*.*CLCe*), with deduced proteins of 700 to 1,031 amino acids. The predicted MWs ranged from 74.03kDa (*BraA1*.*CLCe*) to 110.71 kDa (*BolC1*.*CLCe*), which coincided with protein sizes. The pIs of the CLC proteins varied from 5.18 to 8.86, but most were above 7.0; exceptions were *CLCe* and *CLCf*. Half of the CLC proteins (23) showed instability indices (IIs) < 40.0, whereas the other half presented weak stabilities with IIs > 40.0. The GRAVY values of all *CLCs*, defined as the hydropathy values of all amino acids divided by the protein length, ranged from 0.019 (*BnaC6*.*CLCf*-1) to 0.468 (*BnaA8*.*CLCg*-2), indicating that all *CLC* genes in *Brassica* species are hydrophobic. Most of the *CLC* genes showed similar physicochemical parameters, except for the *CLCf* subfamily, whose GRAVY value was lower than 0.1.

### Evolutionary selection pressures on the CLC proteins of *Brassica* species

**Fig 5:**
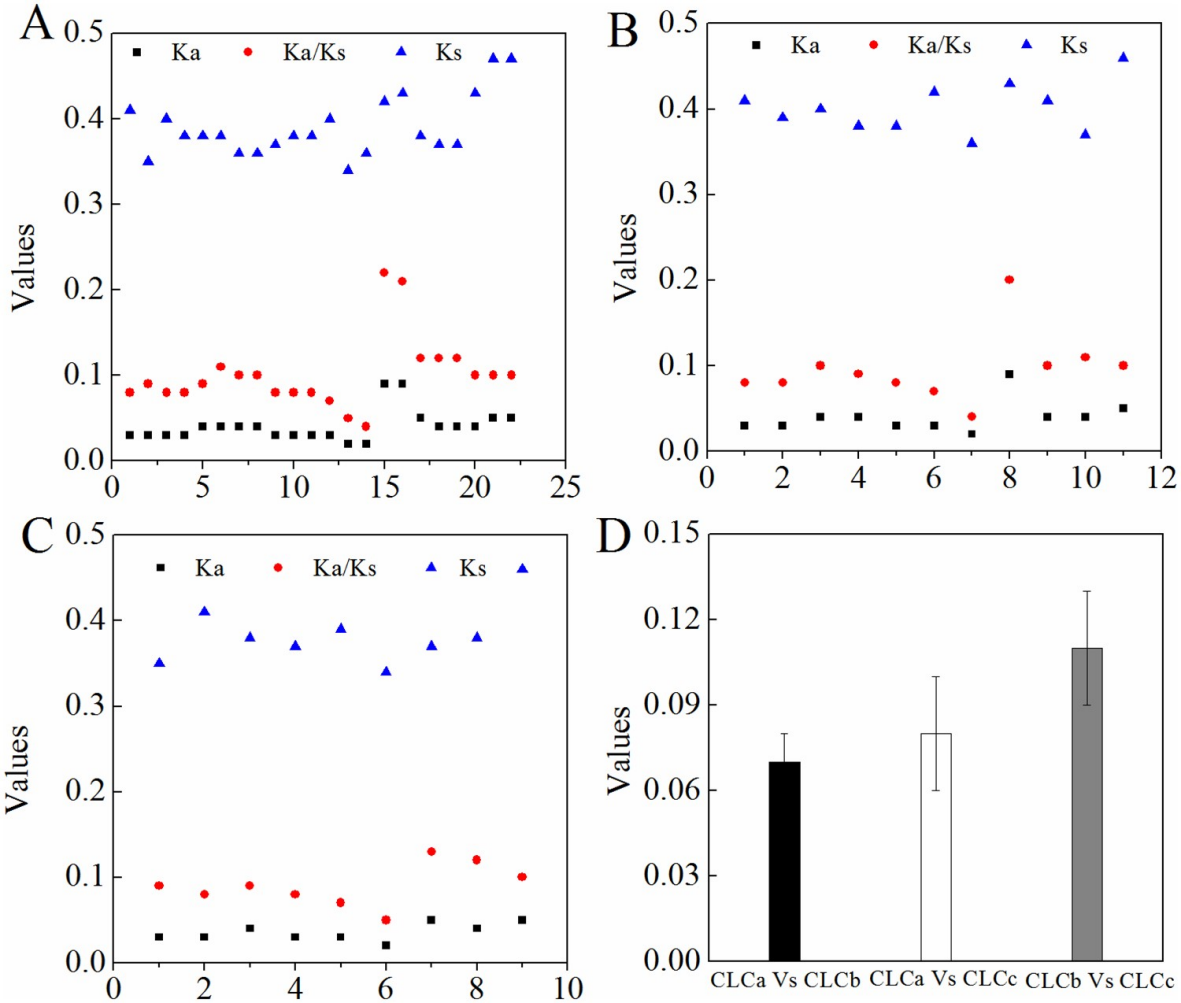
The synonymous substitution rates (Ks) and non-synonymous substitution rates (Ka) of the *CLC* family proteins in Brassica crops. The values of Ks, Ka and Ka/Ks in *B*. *rapa* (A), *B*. *oleracea* (B) and *B*. *napus* (C). The DIVERGE v. 3.0 program was used to detect the type-II functional divergence of the homologs located in the tonoplast in *B*. *napus* (D).

The Ks and Ka values are used to explore the mechanism of gene divergence after duplication [56]. To reveal the selective forces acting on the CLC proteins in *Brassica* species, we calculated the Ka/Ks for orthologous *CLC* gene pairs between the genomes of *Brassica*s pecies and *A*. *thaliana*. Generally, Ka/Ks > 1.0 indicates positive selection, Ka/Ks = 1.0 denotes neutral selection, and Ka/Ks < 1.0 shows negative or purifying selection [56]. As shown in Fig 5A–5C and S2 Table, the Ka/Ks ratios of the *CLC* genes in *Brassica* species were < 0.3. This suggested that a strong purifying selection pressure might have acted on the *CLC* genes of *Brassica* species to maintain gene function. To further investigate the functional divergence of *BnaCLCs*, the DIVERGE 3.0 program was used to estimate the type-II functional divergence (for eight or more homologs) of the *BnaCLC* homologs mainly located in the tonoplast. The coefficient of type-II functional divergence, θ_II_, represents the level of gene functional divergence; it equals 0 when there is no type-II functional divergence and it equals 1 when divergence is very strong [57]. The results revealed that *BnaCLCa*, *BnaCLCb*, and *BnaCLCc* experienced a relatively strong functional divergence, as all of their θ_II_ coefficients were >0 (Fig 5D).

### Identification of gene structure and CREs in the promoters of the *BnaCLC* family genes

**Fig 6:**
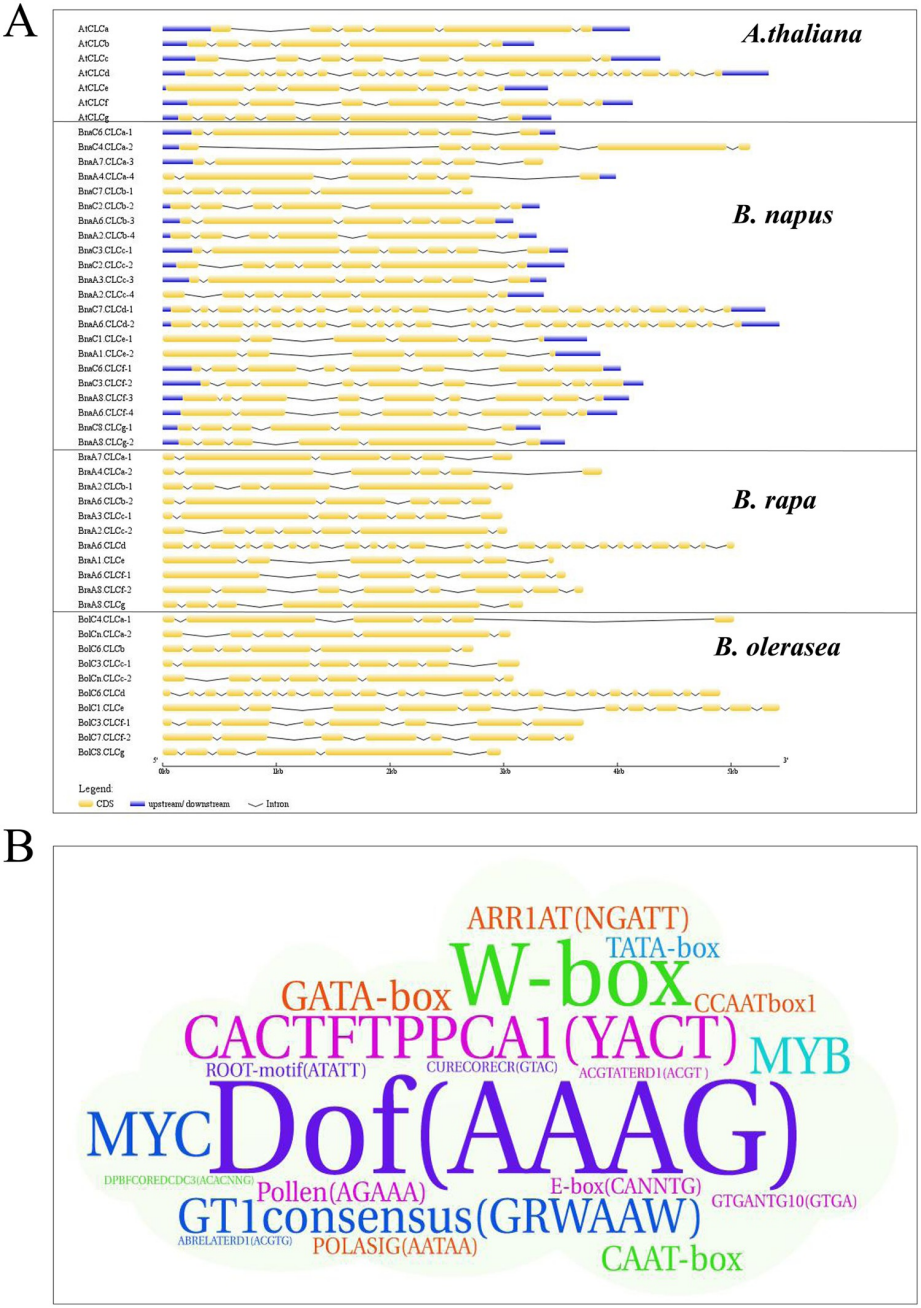
Gene structures and identification of the putative cis-acting regulatory elements (CREs) of the *CLC* family genes in *B*. *napus*. The exon-intron structures of the *CLCs* were determined by the alignments of coding sequences with corresponding genomic sequence (A). The yellow boxes represent exons, blue boxes indicate upstream or down- stream, and the lines represent introns. The diagram is obtained using GSDS Web server. Over-presentation of the CREs in the promoters of the *BnaCLC* family genes is delineated by the Word Art program (B). The bigger the font size is, the larger the CRE number is.

The number and organization of exon-intron structures are typical evolutionary imprints within certain gene families [57]. Therefore, the exon-intron structures of the *CLC* family genes from *Brassica* species were determined by aligning CDS with corresponding genomic sequences, which revealed that the exon-intron structures of the *CLC* genes in *Brassica* species were similar to those of their homologs in *A*. *thaliana*. Most of the *CLC* genes were disrupted by five to eight introns, the exception was the *CLCd* subfamily with 22 introns (Fig 6A). In *B*. *napus*, four *BnaCLC* subfamilies presented five introns, namely *BnaCLCa*/*b*/*e*/*g*. Four genes in the *BnaCLCc* subfamily contained six introns. The *BnaCLCf* subfamily also comprised four genes, but the number of introns varied. Specifically, *BnaCLCf*-1 and *BnaCLCf*-4 had seven introns, whereas *BnaCLCf*-2 and *BnaCLCf*-3 were disrupted by eight introns. The most unique gene in the *BnaCLC* family was *BnaCLCds*, which comprised two genes containing 22 introns.

Transcription factors (TFs) can bind to CREs in the promoters of the genes they regulate and play vital roles in the transcriptional regulation of plant growth [58]. To further understand the transcriptional regulation of CREs in *BnaCLC* genes, we analyzed the CREs present in their promoter sequences (Fig 6B). Among the identified CREs, the DNA-binding One Zinc Finger (Dof)-type CRE was the most enriched (Fig 6B). It has been reported that Dof-type CREs are important in the transcriptional regulation of plant growth under N supply stress [59]. Moreover, excluding the common CREs, such as TATA-box, CAAT-box, and light-responsive elements (e.g., GATA-box and GT1CONSENSUS) [60], some phytohormone responsive elements were identified. These included ARR1AT (NGATT), cytokinin-responsive elements, and abscisic acid-responsive elements, such as ABRELATERD1, ACGTATERD1, MYB, and MYC [61]. The copper-responsive element (CURE) was the only mineral nutrient-responsive element detected in the *BnaCLC* genes.

### Transcriptional profiling and identification of core *BnaCLCs* in response to different NO_3_^-^ levels

**Fig 7:**
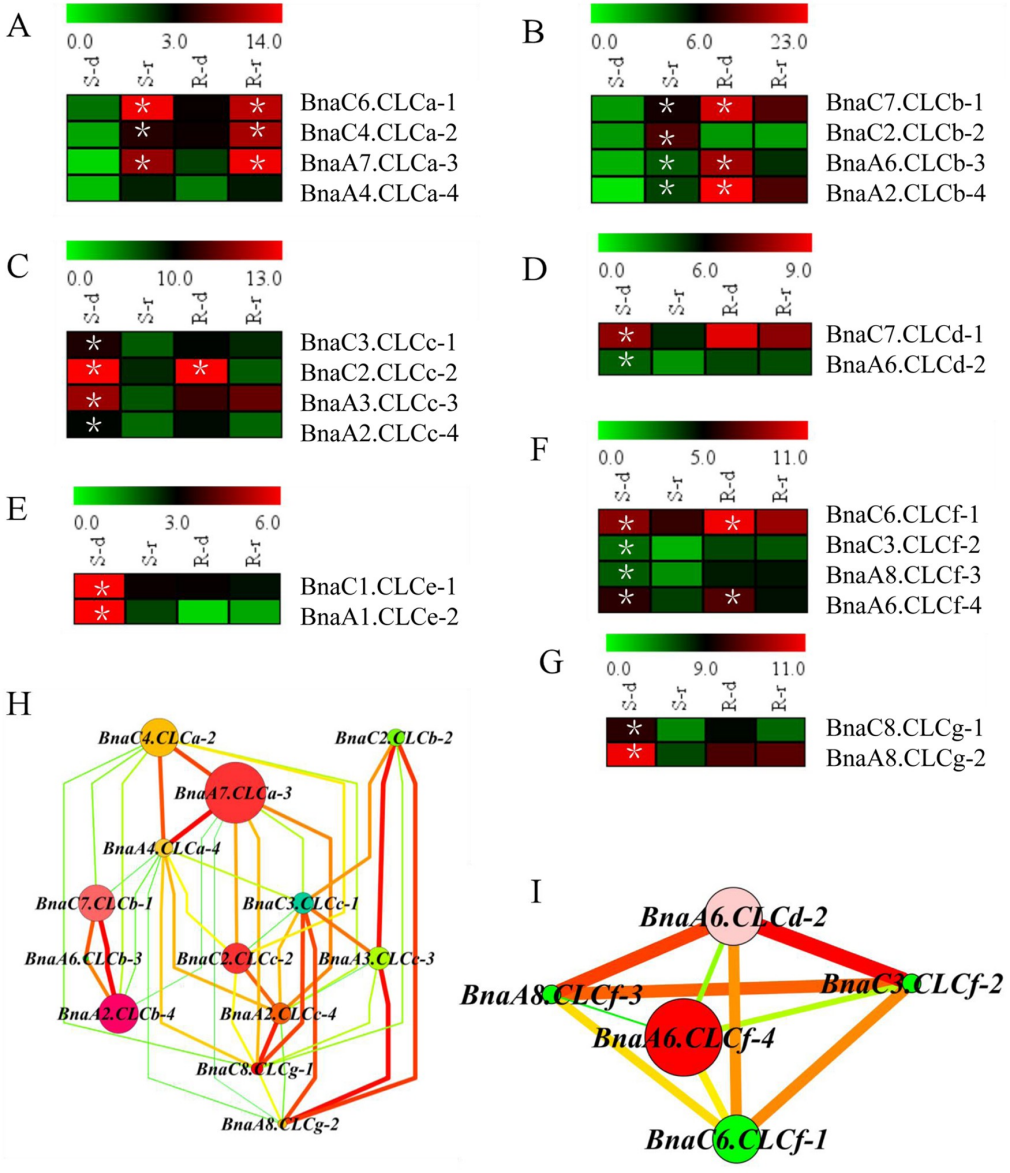
Expression profiling of the BnaCLCs in response to NO_3_^-^ depletion and resupply in *B*. *napus* (A-G). The shoots (S) and roots (R) were sampled separately for RNA seq. S-d shows shoot sample under NO_3_^-^-depletion treatment, and S-r indicates shoot sample under NO_3_^-^-resupply treatment. R-d represents root sample under NO_3_^-^-depletion treatment, and R-r indicates root sample under NO_3_^-^-resupply treatment. The asterisks denote significant differences at *P* < 0.05. Co-expression network analysis of the *BnaCLCs* (H, I). Cycle nodes represent genes, and the size of the nodes represents the power of the interaction among the nodes by degree value. Edges between two nodes represent interactions between genes.

A high-throughput RNA-sequencing analysis was performed to investigate the molecular responses of the *BnaCLCs* to different NO_3_^-^ levels. The shoots and roots of ‘XY15’ were sampled under NO_3_^-^-depletion and NO_3_^-^-replenishment conditions. The expression levels of *BnaCLCas* were strongly induced by NO_3_^-^ replenishment in both shoots and roots (Fig 7A). It has been reported that the response of *AtCLCb* is identical to that of *AtCLCa* [20], whereas the response of *BnaCLCbs* to NO_3_^-^ replenishment is different from that of *BnaCLCas*. In the present study, the transcript abundances of *BnaCLCbs* in the shoots were induced by NO_3_^-^, which was different from that in roots (Fig 7B). The other *BnaCLCs*, including *BnaCLCc–g*, were significantly down-regulated by NO_3_^-^ replenishment in the shoots. In the roots, the transcript levels of *BnaC2*.*CLCc*-2, *BnaC6*.*CLCf-1*, and *BnaA6*.*CLCf-4* were significantly down-regulated by resupplying NO_3_^-^, but the others showed no obvious changes after NO_3_^-^ replenishment (Fig 7C–7G).

To assess the core gene(s) among *BnaCLCs*, a gene co-expression network was constructed according to their protein subcellular localization. It has been reported that *AtCLCa*, *AtCLCb*, *AtCLCc*, and *AtCLCg* are localized to the tonoplasts [15, 20, 22, 23]. *BnaA7*.*CLCa-3* was identified as the core member among the four subfamilies, and it was significantly induced by 20-fold and 6-fold in the shoots and roots, respectively, after NO_3_^-^ replenishment (Fig 7H). In *A*. *thaliana*, AtCLCd and AtCLCf are localized to the Golgi membranes [24–25]. The gene co-expression network identified *BnaA6*.*CLCf-4* as the core member in these two subfamilies, and its expression was suppressed by approximately 1.5-fold after resupplying NO_3_^-^ (Fig 7I). AtCLCe is localized to the thylakoid membrane of chloroplasts [26], and two members were identified in the *BnaCLCe* subfamily. *BnaC1*.*CLCe-1* showed higher transcript abundance than *BnaA1*.*CLCe-2*; thus, we deduced it was the core member in this subfamily (Fig 7E).

### Transcriptional responses of the *BnaCLC* family members to Pi depletion

**Fig 8:**
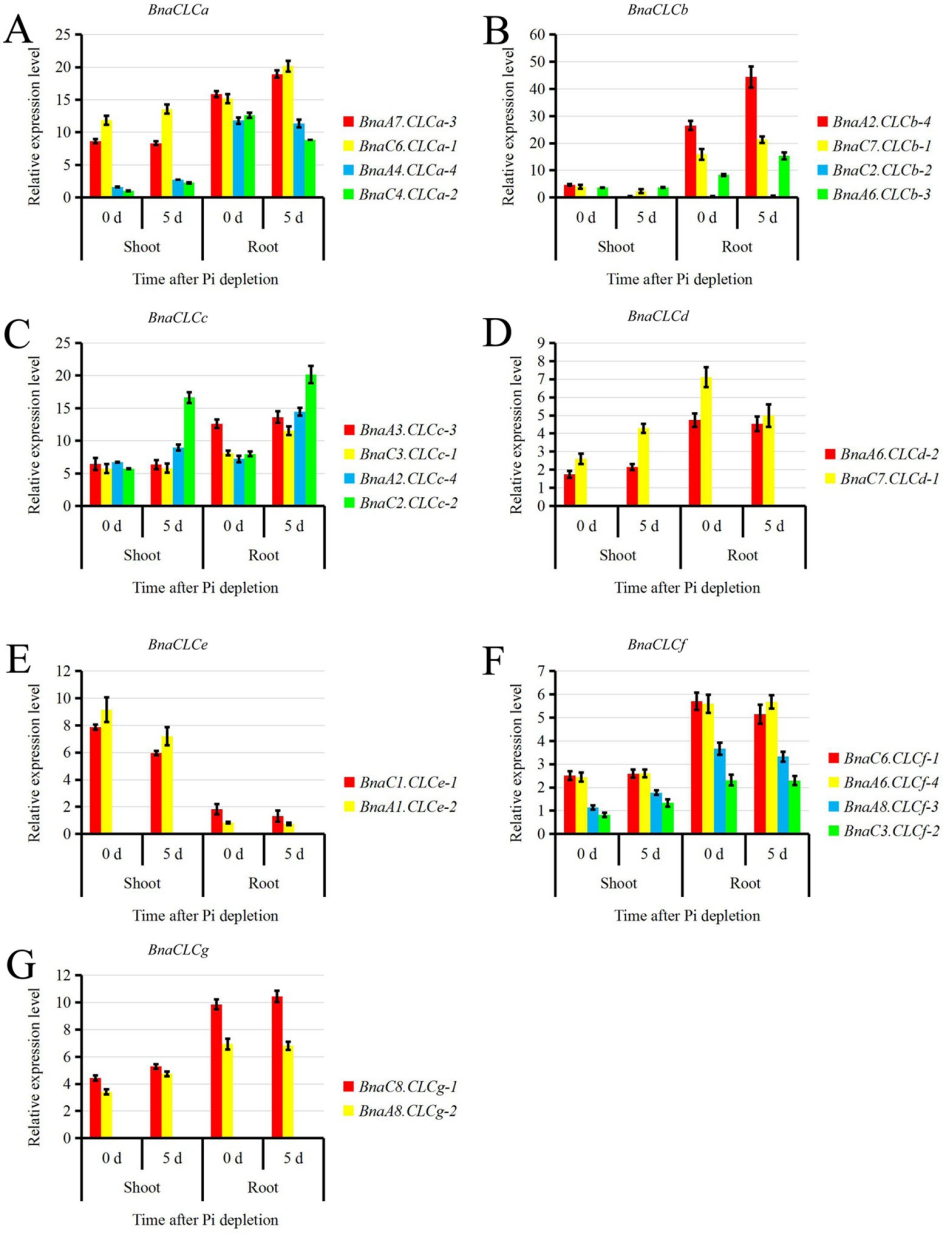
Relative expression of the *BnaCLC* family members under phosphate (Pi) depletion. Relative expression levels of *BnaCLCa* (A), *BnaCLCb* (B), *BnaCLCc* (C), *BnaCLCd* (D), *BnaCLCe* (E), *BnaCLCf* (F), and *BnaCLCg* (G), as revealed by the qRT-PCR assays. For the Pi starvation treatment, the rapeseed seedlings were first grown under 250 μM Pi (KH_2_PO_4_) for 10 d, and then transferred to a Pi-free solution for 5 d. Bars indicate the standard deviation (SD) of three biological replicates.

Elser et al. (2007) showed that plants respond more strongly when N and Pi are added simultaneously than when either of them is added alone [62]. Because the interaction between N and Pi has been widely studied, we investigated the relative expression of the *BnaCLCs* in response to Pi depletion and found that they responded differently (Fig 8). The expression of the core member *BnaA7*.*CLCa-3* in the *BnaCLC* family was up-regulated in the root by Pi depletion (Fig 8A), and the relative expressions of *BnaCLCbs* in the root also significantly increased after Pi depletion, except for *BnaC2*.*CLCb-2* (Fig 8B). Among the *BnaCLCc* subfamily, *BnaC2*.*CLCc-2* and *BnaA2*.*CLCc-4* were induced by Pi depletion both in shoots and roots (Fig 8C). However, *BnaCLCes* were down-regulated whereas *BnaCLCgs* were up-regulated by Pi depletion in both shoots and roots (Fig 8E and 8G).

### Transcriptional responses of the *BnaCLC* family members to Cd toxicity

**Fig 9:**
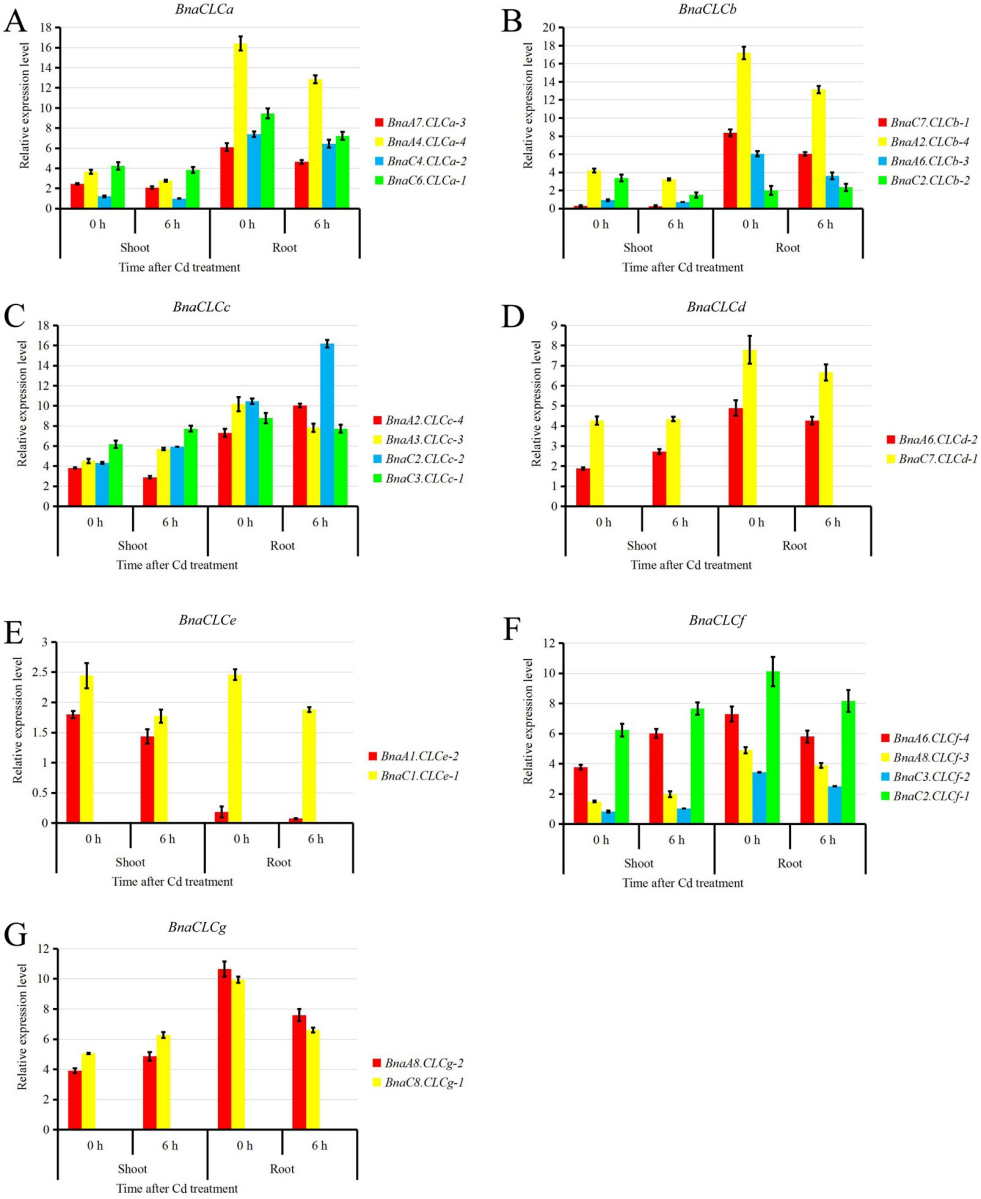
Relative expression of the *BnaCLC* family members under cadmium (Cd) toxicity. Relative expression levels of *BnaCLCa* (A), *BnaCLCb* (B), *BnaCLCc* (C), *BnaCLCd* (D), *BnaCLCe* (E), *BnaCLCf* (F), and *BnaCLCg* (G), as revealed by the qRT-PCR assays. For the Cd toxicity treatment, the rapeseed seedlings were first grown under a Cd-free solution for 10 d, and then transferred to 10 μM CdCl_2_ for 6 h. Bars indicate the standard deviation (SD) of three biological replicates.

Previous studies demonstrated that *AtCLCs* play important roles in the regulation of abiotic and biotic stresses [22, 23, 63]. Thus, we investigated the relative expression of *BnaCLCs* under Cd stress and found that the relative expression of the *BnaCLCa* and *BnaCLCb* members was down-regulated by Cd stress in the root but no significant change was observed in the shoot (Fig 9A and 9B). Whereas *BnaC3*.*CLCc-1* and *BnaA3*.*CLCc-3* were slightly down-regulated, *BnaC2*.*CLCc-2* and *BnaA2*.*CLCc-4* were up-regulated by Cd in the root (Fig 9C); *BnaC3*.*CLCc-1*, *BnaC2*.*CLCc-2*, and *BnaA3*.*CLCc-3* were up-regulated whereas *BnaA2*.*CLCc-4* was down-regulated under Cd stress in the shoot (Fig 9C). The *BnaCLCd*, *BnaCLCf* and *BnaCLCg* subfamilies showed similar responses to Cd stress, as the relative expressions of these genes were up-regulated in the shoot and down-regulated in the root (Fig 9D, 9F and 9G). The *BnaCLCe* genes were suppressed by Cd stress in both shoot and root (Fig 9E).

## Discussion

### Genome-wide characterization of the *CLC* genes in *B*. *napus*

The CLC proteins are first characterized as 2Cl^−^/1H^+^ antiporters specifically involved in chloride transport [16–17]. De Angeli et al. (2006) demonstrated that AtCLCa is a tonoplast-located 2NO_3_^-^/1H^+^ antiporter that participates in the regulation of NO_3_^-^ storage in vacuoles [15]. The CLC proteins can be found in all organisms, and seven *AtCLCs* have been identified in *A*. *thaliana* [12]. Our results revealed higher activity of NR and GS in the *clca-2* mutant, whose N assimilation was thus accelerated, and the NUE was also significantly higher in the *clca-2* mutant (Fig 1). Nitrate is a major N form and it can be reduced to NH_4_^+^ by NR and nitrite reductase (NiR), and then synthesized into amino acids through the GS/glutamine-2-oxoglutarate aminotransferase (GOGAT) pathway [64–65]. More importantly, the activity of both NR and GS can be induced by NO_3_^-^ [66]. Less NO_3_^-^ was transported to the vacuoles of the *clca-2* mutant than to the vacuoles of Ws, and the NO_3_^-^ in the cytoplasm induced the activities of NR and GS. Thus, a higher NUE was found in the*clca-2* mutant [4]. Moreover, high-NUE genotypes showed higher activities of NR and GS, which accelerated NO_3_^-^ assimilation in *B*. *napus* [4, 8]. Therefore, high NR and GS activities contribute to enhance NUE in both *A*. *thaliana* and *B*. *napus*. NO_3_^-^ status affects the activity of NR and GS in plants [66], and it has been reported that the *CLC* genes play vital roles in the regulation of NO_3_^-^ homeostasis. However, the functions of the *CLC* family genes and their effects on NUE were rarely reported in *B*. *napus*. To achieve a better understanding of the biological function of *CLC* genes, we performed a comprehensive analyses of this family in *B*. *napus*. In addition, *B*. *napus* is a main oil crop that originated from the natural hybridization between the intact genomes of *B*. *oleracea* and *B*. *rapa*. Due to the genome complexity and high N demand of *B*. *napus*, it is important to investigate the molecular functions of the *BnaCLC* genes.

In the present study, 22 *CLC* family genes were identified in *B*. *napus* based on the corresponding homologs in *A*. *thaliana* (S1 Table). All these genes were divided into the same seven subfamilies as the *CLCs* in *A*. *thaliana* (i.e., *AtCLCa-g*) (Fig 2), and they were evenly distributed in seven chromosomes of the A_n_ subgenome and seven chromosomes of the C_n_ subgenome (Fig 3A). This further supported the hypothesis that the *B*. *napus* genome results from the hybridization of *B*. *rapa* (A_r_) and *B*. *oleracea* (C_o_) genomes [67]. Genome duplication, segmental duplication, and tandem duplication are the main factors contributing to the expansion of gene families [54, 68]. Our results revealed that whole genome duplication and segmental duplication were the driving forces of the *CLC* gene expansion in *B*. *napus* (Fig 3B). All the *BnaCLCs* genes underwent strong purifying selection, as evidenced by Ka/Ks ratios lower than 1.0 (Fig 4A–4C; S2 Table), which indicated that the *CLC* gene function was highly preserved. Moreover, obvious gene functional divergence occurred among the *CLCa*, *CLCb* and *CLCc* subfamilies in *B*. *napus* (Fig 4D).

### Genome-wide identification and transcriptomics-assisted gene co-expression network analysis characterized the core members of the *BnaCLC* gene family

The compartmentalization of NO_3_^-^ in organelles is critical for plant physiology [4]. It has been reported that several *AtCLCs* are involved in NO_3_^-^ transport into vacuoles, such as *AtCLCa*, *AtCLCb* and *AtCLCc* [15, 20, 21]. Our results revealed that N assimilation was enhanced in the *clca-2* mutants, which contributed to its higher NUE (Fig 1). High-throughput transcriptomics was performed to explore the molecular responses of the *BnaCLCs* to different NO_3_^-^ supply levels (Fig 7). It showed that the transcript abundance of *BnaCLCas* in both shoot and root was significantly enhanced by NO_3_^-^ after N starvation (Fig 7A), which was similar to the response of *AtCLCa* to NO_3_^-^ in *A*. *thaliana* [18]. Gene *CLCb*, a close relative of *CLCa*, was weakly upregulated (1.5–2.0-fold) by NO_3_^-^ [20, 69, 70]. The *BnaCLCb* transcripts were significantly induced and repressed by NO_3_^-^ in the shoot and root of *B*. *napus*, respectively (Fig 7B). However, the other five *BnaCLCs*, namely *BnaCLCc*, *BnaCLCd*, *BnaCLCe*, *BnaCLCf* and *BnaCLCg*, were repressed by NO_3_^-^ replenishment (Fig 7C–7G). In *A*. *thaliana*, decreased expression of *AtCLCc* mRNA was also observed within 0.6 h when Ca(NO_3_)_2_ was supplied to N-starved plants [18]. The different responses of *BnaCLCs* to NO_3_^-^ might be related to anion specificity. For example, *AtCLCa* specifically mediates NO_3_^-^ transport because the proline in its selectivity filter motif GXGIP plays an important role in NO_3_^-^ selectivity [71]. However, *AtCLCc* has a broader anion specificity, including chloride, malate, and citrate [15, 18].

Sets of *BnaCLCs* found in duplicated segments could have redundant/duplicate gene functions; therefore, the core members should be characterized. Based on their different subcellular localizations, gene co-expression analysis identified *BnaA7*.*CLCa-3*,*BnaA6*.*CLCf-4*, and *BnaC1*.*CLCe-1* as the core members of the *BnaCLC* gene family (Fig 7H and 7I). AtCLCa functions as a 2NO_3_^-^/1H^+^ antiporter and plays a vital role in NO_3_^-^ transportation into vacuoles [15], and *AtCLCe* is reported to function in the regulation of photosynthetic electron transport [26]; however, the gene function of *AtCLCf* is still unknown [25]. Therefore, we propose that *BnaA7*.*CLCa-3* is the most important member of the *BnaCLC* gene family. In addition, *BnaA7*.*CLCa-3* response to NO_3_^-^ was similar to that of A*tCLCa* (Fig 7A), suggesting that *BnaA7*.*CLCa-3* might play a crucial role in the transportation of NO_3_^-^ and regulation of NUE in *B*. *napus*.

### Potential regulation of *BnaCLCs* and their contribution to NUE improvement and tolerance to biotic or abiotic stresses

We identified a set of CREs in the promoters of the *CLC* family genes in *B*. *napus* (Fig 6B). The most abundant were Dof, W-box, MYB and GATA-box. The Dof, GATA-box, and MYB TFs have been implicated in the molecular responses of plants to N status [72–74]. For instance, overexpression of *ZmDOF1* resulted in increased N content and improved growth in transgenic *A*. *thaliana* plants under low N conditions [75–76]. Rice *OsDOF18*, the most homologous gene to *ZmDOF1* [77–78], modulates ammonium uptake by inducing ammonium transporter genes [74]. W-box (AGCT) is an emerging player in plant signaling and has been reported to play important roles in plant responses to various biotic and abiotic stresses [79]. Our results revealed that W-box was one of the most abundant CREs in the promoter regions of the *CLC* family genes in *B*. *napus* (Fig 6B). Previous studies have shown that *AtCLCs* play key roles in responses to biotic and abiotic stresses. For example, *AtCLCc* is involved in stomatal movement and contributes to salt tolerance [22]. Although *AtCLCg* shares a high degree of identity (62%) with *AtCLCc*, and both are important for tolerance to excess chloride, their functions are not redundant [23]. Furthermore, *AtCLCd* negatively regulates pathogen-associated molecular pattern triggered immunity [63]. Our results showed that the *BnaCLCs* showed distinct responses to nutrient depletion and heavy metal stress. N and P are essential nutrients for plant growth and the interaction between them was thoroughly discussed by Agren et al. (2012) [80]. In addition, Li et al. (2009) revealed that low-Pi stress down-regulated the genes coding for NR and GS, which weakened N assimilation [81]. Our results revealed that most of the *BnaCLCs* were up-regulated by Pi depletion (Fig 8). The *CLC* genes play vital roles in NO_3_^-^ storage and its function loss promoted N assimilation [4]. Up-regulation of the *BnaCLCs* under Pi depletion might increase NO_3_^-^ storage in vacuoles, which results in less NO_3_^-^ allocated to the cytoplasm, and NO_3_^-^ storage in the vacuole weakens its assimilation. Notably, low-Pi stress influenced N assimilation through different pathways. In addition, the *BnaCLCs* showed distinct expression patterns under Cd toxicity, and thus might play pivotal roles in the regulation of Cd detoxification (Fig 9). The vacuole occupies 60–95% of the mature plant cell, and itis not only the site for storing nutrients, but also an important organelle for sequestering toxic heavy metals, such as Cd [2, 82, 83]. The proton pumps in the tonoplast, vacuolar H^+^-pyrophosphatase (V-PPase) and the vacuolar H^+^-ATPase (V-ATPase), are responsible for establishing ΔH^+^ between cytoplasm and vacuoles [84–85], providing the driving force for ion transmembrane transport. AtCLCa is a tonoplast-localized 2NO_3_^-^/H^+^ antiporter that is involved in the regulation of NO_3_^-^ sequestration into vacuoles [15]. Vacuolar compartmentalization is central for heavy metal homeostasis. It depends on two vacuolar pumps (V-ATPase and V-PPase) and on a set of tonoplast transporters, which are directly driven by proton motive force and primary ATP-dependent pumps [83]. Hence, both NO_3_^-^ and Cd sequestration into vacuoles rely on the proton motive force established by V-ATPase and V-PPase. When NO_3_^-^ storage is decreased, lower proton driving force is required. Our results showed that the *BnaCLCs* were down-regulated by Cd (Fig 9), which decreased NO_3_^-^ transportation into vacuole. Consequently, the proton driving force might diverted to transport Cd into the vacuole and improve Cd tolerance of *B*. *napus*. However, further research is required to reveal the functions of *BnaCLCs* in Cd detoxification.

## Conclusion

Through genome-wide analysis of the *CLC* family genes in *B*. *napus*, a total of 22 *BnaCLCs* were identified in the rapeseed genome. We found that genome-wide duplication and segmental duplication of the *CLC* genes contributed to a relatively large *CLC* gene family in *B*. *napus*. These genes showed high orthologous relationships with corresponding *AtCLC* homologs, and they were classified into seven subgroups. *AtCLCa* has been reported to transport NO_3_^-^ into vacuoles. We found that the *clca-2* mutant showed enhanced N assimilation ability and high NUE. Therefore, to further explore the potential roles of *BnaCLCs* in vacuolar NO_3_^-^ transport, a high-throughput transcriptomics analysis was performed. The results revealed that different *BnaCLCs* showed distinct responses to NO_3_^-^ levels. Nevertheless, the general responses of *BnaCLCa* and *BnaCLCc* to NO_3_^-^ replenishment were the same as *AtCLCa* and *AtCLCc*. In addition, gene co-expression analysis revealed that *BnaA7*.*CLCa-3* was the core member of the *BnaCLC* family. Its expression was up-regulated when exposed to both NO_3_^-^ resupply and Pi depletion, and it was down-regulated by Cd toxicity. Moreover, two enriched CREs, including Dof and W-box, were abundant in the promoter regions of *BnaCLCs*, which might contribute to NUE improvement and stress tolerance. These findings provide a fundamental basis for improving crops’ NUE and tolerance to diverse stresses through genetic engineering of the *CLC* genes.

## Supporting information

S1 Table Comparative analysis of the *CLC* family genes in Brassica species. (DOCX)

S2 Table Synonymous rate (Ks) and non-synonymous rate (Ka) of nucleotide substitution in the *CLC* family genes of Brassica species. (DOCX)

S3 Table RNA-seq data of the *BnaCLCs* in response to NO_3_^-^ depletion and resupply in *B*. *napus*. (RAR)

S1 Fig Short amino acid sequences of the 10 conserved motifs and three common motifs in the *CLC* family genes. The 10 conserved motifs predicted by the MEME program (A). Characterization of the three common motifs (motifs 4/7/10) in the *CLC* proteins of *A*. *thaliana* and *Brassica* species (B), as obtained in Weblogo. The larger the font, the more conserved is the motif.
(DOCX)

## Abbreviations

ANOVA: analysis of variance
BLAST: basic local alignment search tool
Cd: cadmium
CDS: coding sequences
CLC: *Chloride Channel*
CREs: *cis*-acting regulatory elements
CURE: copper-responsive element
Dof: DNA-binding One Zinc Finger
GS: glutamine synthetase
HSD: honestly significant difference
IIs: instability indices
MWs: molecular weights
N: Nitrogen
NCBI: National Center for Biotechnology Information
NO_3_^-^: inorganic nitrate
NR: nitrate reductase
NRT: nitrate transporter
NUE: N use efficiency
Pi: inorganic phosphate
PIs: isoelectric points
TFs: transcription factors
V-ATPase: vacuolar H^+^-ATPase
V-PPase: vacuolar H^+^-pyrophosphatase
VSC: vacuolar sequestration capacity
