# Supplementary material for: Genome-scale characterization of the vacuole nitrate transporter Chloride Channel (CLC) genes and their transcriptional responses to diverse nutrient stresses in allotetraploid rapeseed
Source: PLoS One. 2018 Dec 20;13(12):e0208648. doi: 10.1371/journal.pone.0208648 (PMC6301700; doi:10.1371/journal.pone.0208648)
Supplement: S1 Table — (DOCX) [file pone.0208648.s001.docx]

**S1 Table Comparative analysis of the *CLC* family genes in *Brassica* species**

| Species name | Genome size (Mb) | *CLCa* | *CLCb* | *CLCc* | *CLCd* | *CLCe* | *CLCf* | *CLCg* |
| --- | --- | --- | --- | --- | --- | --- | --- | --- |
| *Brassica napus* | 1345 | 4 | 4 | 4 | 2 | 2 | 4 | 2 |
| *Brassica rapa* | 485 | 2 | 2 | 2 | 1 | 1 | 2 | 1 |
| *Brassica oleracea* | 630 | 2 | 1 | 2 | 1 | 1 | 2 | 1 |
