# Supplementary material for: Genome-scale characterization of the vacuole nitrate transporter Chloride Channel (CLC) genes and their transcriptional responses to diverse nutrient stresses in allotetraploid rapeseed
Source: PLoS One. 2018 Dec 20;13(12):e0208648. doi: 10.1371/journal.pone.0208648 (PMC6301700; doi:10.1371/journal.pone.0208648)
Supplement: S2 Table — (DOCX) [file pone.0208648.s002.docx]

**S2 Table Synonymous rate (Ks) and non-synonymous rate (Ka) of nucleotide substitution in the *CLC* family genes of *Brassica* species.**

| Name | Ka | Ks | Ka/Ks | P-Value (Fisher) | Divergence time (Mya) |
| --- | --- | --- | --- | --- | --- |
| *BnaC6.CLCa-1* | 0.031 | 0.414 | 0.075 | 2.03E-71 | 13.79 |
| *BnaC4.CLCa-2* | 0.032 | 0.346 | 0.091 | 4.50E-58 | 11.55 |
| *BnaA7.CLCa-3* | 0.030 | 0.398 | 0.075 | 3.14E-69 | 13.27 |
| *BnaA4.CLCa-4* | 0.031 | 0.382 | 0.080 | 2.34E-66 | 12.74 |
| *BraA7.CLCa-1* | 0.030 | 0.405 | 0.075 | 4.43E-70 | 13.51 |
| *BraA4.CLCa-2* | 0.030 | 0.385 | 0.078 | 1.35E-67 | 12.83 |
| *BolC4.CLCa-1* | 0.032 | 0.346 | 0.091 | 4.50E-58 | 11.55 |
| *BolCn.CLCa-2* | 0.031 | 0.414 | 0.075 | 2.03E-71 | 13.79 |
| *BnaC7.CLCb-1* | 0.035 | 0.378 | 0.091 | 1.45E-63 | 12.61 |
| *BnaC2.CLCb-2* | 0.041 | 0.377 | 0.109 | 1.87E-59 | 12.58 |
| *BnaA6.CLCb-3* | 0.036 | 0.364 | 0.099 | 4.28E-60 | 12.13 |
| *BnaA2.CLCb-4* | 0.036 | 0.364 | 0.099 | 4.28E-60 | 12.13 |
| *BraA2.CLCb-1* | 0.040 | 0.399 | 0.100 | 4.65E-64 | 13.29 |
| *BraA6.CLCb-2* | 0.035 | 0.376 | 0.092 | 1.39E-63 | 12.54 |
| *BolC6.CLCb* | 0.035 | 0.381 | 0.092 | 2.11E-63 | 12.69 |
| *BnaC3.CLCc-1* | 0.030 | 0.367 | 0.081 | 2.48E-66 | 12.25 |
| *BnaC2.CLCc-2* | 0.029 | 0.383 | 0.077 | 2.74E-70 | 12.77 |
| *BnaA3.CLCc-3* | 0.030 | 0.380 | 0.079 | 5.06E-69 | 12.68 |
| *BnaA2.CLCc-4* | 0.029 | 0.400 | 0.072 | 1.83E-74 | 13.32 |
| *BraA3.CLCc-1* | 0.029 | 0.377 | 0.078 | 5.78E-69 | 12.57 |
| *BraA2.CLCc-2* | 0.029 | 0.423 | 0.068 | 3.80E-79 | 14.10 |
| *BolC3.CLCc-1* | 0.030 | 0.367 | 0.081 | 2.48E-66 | 12.25 |
| *BolCn.CLCc-2* | 0.029 | 0.389 | 0.074 | 7.11E-72 | 12.97 |
| *BnaC7.CLCd-1* | 0.015 | 0.343 | 0.045 | 2.56E-73 | 11.43 |
| *BnaA6.CLCd-2* | 0.015 | 0.355 | 0.043 | 7.23E-75 | 11.82 |
| *BraA6.CLCd* | 0.015 | 0.355 | 0.043 | 1.17E-74 | 11.82 |
| *BolC6.CLCd* | 0.015 | 0.339 | 0.046 | 1.02E-72 | 11.29 |
| *BnaC1.CLCe-1* | 0.093 | 0.421 | 0.221 | 3.81E-35 | 14.05 |
| *BnaA1.CLCe-2* | 0.089 | 0.427 | 0.210 | 4.15E-37 | 14.22 |
| *BraA1.CLCe* | 0.085 | 0.430 | 0.198 | 2.16E-38 | 14.33 |
| *BnaC6.CLCf-1* | 0.045 | 0.384 | 0.116 | 3.91E-55 | 12.80 |
| *BnaC3.CLCf-2* | 0.044 | 0.371 | 0.118 | 1.01E-52 | 12.36 |
| *BnaA8.CLCf-3* | 0.043 | 0.368 | 0.116 | 7.82E-53 | 12.27 |
| *BnaA6.CLCf-4* | 0.043 | 0.425 | 0.101 | 7.52E-64 | 14.17 |
| *BraA6.CLCf-1* | 0.042 | 0.406 | 0.103 | 6.30E-61 | 13.55 |
| *BraA8.CLCf-2* | 0.041 | 0.373 | 0.111 | 2.29E-55 | 12.43 |
| *BolC3.CLCf-1* | 0.048 | 0.367 | 0.130 | 3.02E-50 | 12.22 |
| *BolC7.CLCf-2* | 0.044 | 0.382 | 0.115 | 3.23E-55 | 12.72 |
| *BnaC8.CLCg-1* | 0.047 | 0.473 | 0.100 | 2.20E-68 | 15.76 |
| *BnaA8.CLCg-2* | 0.047 | 0.465 | 0.100 | 9.93E-69 | 15.51 |
| *BraA8.CLCg* | 0.046 | 0.460 | 0.100 | 1.88E-68 | 15.32 |
| *BolC8.CLCg* | 0.047 | 0.461 | 0.103 | 1.83E-66 | 15.36 |

Note: Divergence time was calculated by the formula T = Ks/2λ (λ = 1.5 × 10^-8^ for Brassicaceae)
