# Supplementary material for: Genome-scale characterization of the vacuole nitrate transporter Chloride Channel (CLC) genes and their transcriptional responses to diverse nutrient stresses in allotetraploid rapeseed
Source: PLoS One. 2018 Dec 20;13(12):e0208648. doi: 10.1371/journal.pone.0208648 (PMC6301700; doi:10.1371/journal.pone.0208648)
Supplement: S1 Fig — The 10 conserved motifs predicted by the MEME program (A). Characterization of the three common motifs (motifs 4/7/10) in the CLC proteins of A. thaliana and Brassica species (B), as obtained in Weblogo. The larger the font, the more conserved is the motif. (DOCX) [file pone.0208648.s004.docx]

**S1 Fig. Short amino acid sequences of the 10 conserved motifs and three common motifs in the *CLC* family genes.** The 10 conserved motifs predicted by the MEME program (A). Characterization of the three common motifs (motifs 4/7/10) in the *CLC* proteins of *A. thaliana* and *Brassica* species (B), as obtained in Weblogo. The larger the font, the more conserved is the motif.
